# Species distribution modeling reveals strongholds and potential reintroduction areas for the world’s largest eagle

**DOI:** 10.1371/journal.pone.0216323

**Published:** 2019-05-13

**Authors:** Everton B. P. Miranda, Jorge F. S Menezes, Camila C. L. Farias, Charles Munn, Carlos A. Peres

**Affiliations:** 1 Universidade Estadual de Mato Grosso, Alta Floresta, Mato Grosso, Brazil; 2 School of Life Sciences, University of KwaZulu-Natal, Pietermaritzburg, South Africa; 3 Programa de Pós-graduação em Ecologia, IB, Universidade de Brasília, Brasília, Distrito Federal, Brazil; 4 Mitrani Department of Desert Ecology, Ben Gurion University of the Negev, Beersheba, Israel; 5 Faculdade de Medicina Veterinária, Universidade Federal de Mato Grosso, Cuiabá, Mato Grosso, Brazil; 6 SouthWild, Várzea Grande, Mato Grosso, Brazil; 7 Center for Ecology, Evolution and Conservation, School of Environmental Sciences, University of East Anglia, Norwich, United Kingdom; Michigan State University, UNITED STATES

## Abstract

The highly interactive nature of predator-prey relationship is essential for ecosystem conservation; predators have been extirpated, however, from entire ecosystems all over the Earth. Reintroductions comprise a management technique to reverse this trend. Species Distribution Models (SDM) are preemptive tools for release-site selection, and can define levels of habitat quality over the species distribution. The Atlantic Forest of South America has lost most of its apex predators, and Harpy Eagles *Harpia harpyja*—Earth’s largest eagle—are now limited to few forest pockets in this domain. Harpy Eagles are supposedly widespread in the Amazon Forest, however, where habitat loss and degradation is advancing at a rapid pace. We aim to describe the suitability of threatened Amazonian landscapes for this eagle. We also aim to assess the suitability of remaining Atlantic Forest sites for Harpy Eagle reintroductions. Here we show that that considerable eagle habitat has already been lost in Amazonia due to the expansion of the “Arc of Deforestation”, and that Amazonian forests currently represent 93% of the current distribution of the species. We also show that the Serra do Mar protected areas in southeastern Brazil is the most promising region for Harpy Eagle reintroductions in the Atlantic Forest. Reintroduction and captive breeding programs have been undertaken for Harpy Eagles, building the technical and biological basis for a successful restoration framework. Our distribution range for this species represents a 41% reduction of what is currently proposed by IUCN. Furthermore, habitat loss in Amazonia, combined with industrial logging and hunting suggest that the conservation status of this species should be reassessed. We suggest researchers and conservation practitioners can use this work to help expand efforts to conserve Harpy Eagles and their natural habitats.

## Introduction

Extensive losses of apex predators is a pervasive conservation problem in ecosystems around the world [1]. Since the appearance of hominids ~2 million years ago, competition for wild prey, fear of direct attack on humans, and predation on domestic animals has led to the decimation of predator populations [2–4]. The subsequent cascading effects of predator-free populations of herbivores on plant communities can thus damage both natural vegetation and associated biodiversity [5–7]. These issues have placed predators near the top of the conservation biology agenda [8,9], and reintroductions have emerged as one of the main conservation tools to reverse these trends [10,11].

**Fig 1:**
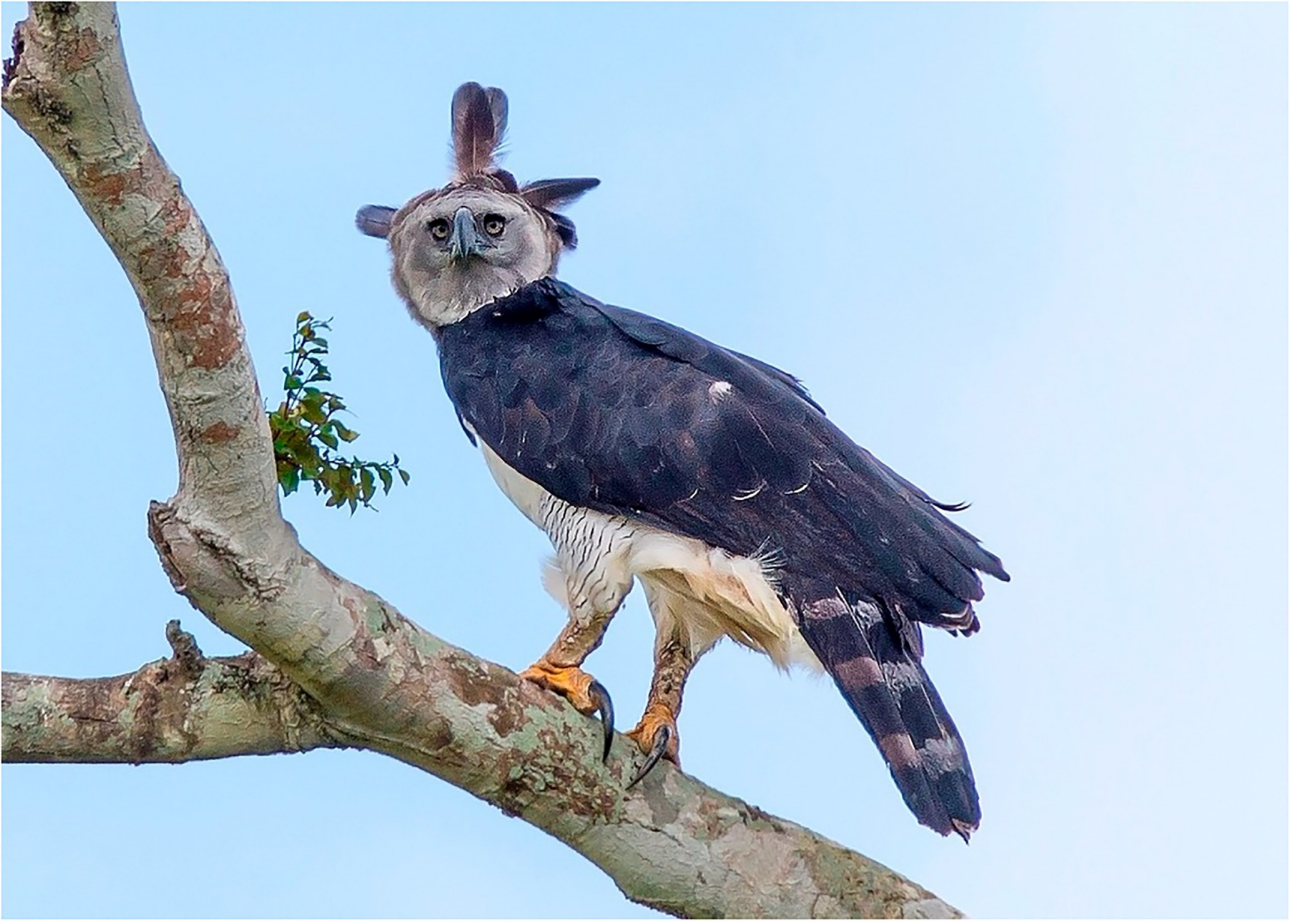
Harpy Eagle *Harpia harpyja* adult female perched in the Atlantic Forest of Sooretama Reserve, state of Espirito Santo, Brazil [25].

Few living predators are as quintessential creatures of legend as the Harpy Eagle (Fig 1, *Harpia harpyja*; [12,13]). Averaging 6.6 kg, the Harpy Eagle is the largest extant raptor on Earth, and is surpassed in size by only the extinct, island-living Haast Eagle (*Harpagornis moorei*; [14]), which humans wiped out from New Zealand’s South Island. The Harpy Eagle is a forest species with the lowest reproductive rate of any living bird, producing a single offspring every 30–36 months [15,16]. Harpies have been persecuted over their entire range [17–20], and their feathers and talons are ubiquitous ornaments, with feathers often part of Amerindian arrows and headdresses [21,22]. Live eagles are also captured and kept by Amerindians as sources of feathers ([21]; personal observation). These factors, combined with habitat loss and degradation through logging, have already led to the rarity or extirpation of Harpies in much of their geographic distribution [23], especially in the Brazilian Atlantic Forest biodiversity hotspot [24].

The Atlantic Forest has suffered widespread losses of top predators [26]. Jaguars *Panthera onca* survive in the Atlantic Forest in only eight forest pockets, with a total estimated remaining population of only 300 individuals [27]. Relict populations of Harpy Eagles in the Atlantic Forest are currently known from around 10 breeding pairs and a few scattered individuals [28–31]. Harpy Eagles have been shown to exert strong behavioral and demographic control over their prey species [6,32]. In the absence of Harpies, prey populations often experience unfettered growth [33]. Consequently, they can be described as a keystone predator. Cascading consequences rising from the absence of Harpy Eagles are known to affect prey species. For instance, hyper-abundant populations of Black Capuchin Monkeys (*Sapajus nigritus*) cause high mortality of an arborescent palm (*Euterpe edulis*) of the Atlantic Forest because they rip out and eat the apical meristem, known as “palmito” [34,35]. This palm species is itself a threatened key species of the Atlantic Forest, and benefits many species of frugivores by producing year-round infructescences, which are particularly important during the annual period of general, community-wide fruit scarcity [36]. Throughout the entire distribution of the Harpy Eagle, various species of capuchins represent the second most common primate prey [37]. Restoring Harpy Eagle populations would restore balanced communities in the ecosystem by reducing capuchin monkey densities, thereby preventing harmful plant-herbivore interactions. Management guidelines could therefore benefit considerably from prioritizing which forest regions are most suitable for restoration of Harpy Eagle populations. Species Distribution Modeling (SDM; *sensu* [38]) can help obtain those answers.

Harpy Eagles are currently considered Near Threatened by the International Union for Conservation of Nature, IUCN [39]. Whereas the species has vanished throughout much of its historical distribution [23], its widespread occurrence in vast tracts of Amazonian forests prevents Harpies from being listed in a higher threat category [39]. Meanwhile, questions remain about the quality of the supposedly homogeneously-pristine tracts of eagle habitat across Amazonia. Improving knowledge on this topic has high value for conservation, since the ever-expanding cattle ranching frontier in a region of the southern Amazon known as the “Arc of Deforestation” has rapidly converted vast tracts of Amazonian forests into pasture and soy fields [40]. This forest destruction has led to loss of genetic diversity in Harpy Eagles [41]. SDMs could provide an improved basis for discussions about Harpy eagle distribution in neotropical forests as well as in fringe forest habitats such as the Brazilian Cerrado and Pantanal wetlands, thereby helping delineate the biogeographic boundaries of future reintroduction programs. Therefore, building SDMs for Harpy Eagles is central to a sound conservation strategy for this apex predator.

A significant challenge to building a Harpy Eagle SDM is that to produce a robust result, one requires a significant amount of widely distributed geographic records [42]. Existing records, however, might be either too few or too patchy to produce a reliable SDM for such an elusive species. Finding Harpy Eagle nests has proven so difficult that the discovery of a single nest often sparks widespread excitement among ornithologists [43–45]. Furthermore, the few museum records of this species are severely restricted in range [46]. Finally, most museum skins include no data on the breeding status of the specimens, information that can greatly improve the quality of SDMs. We further highlight the unmet potential of the only attempt to compile a sufficient number of geographic records to unravel the Harpy Eagle distribution [23]; but this study failed to produce even the simplest map. Although two different, long-term Harpy Eagle field projects have each located more than a hundred nests, they have failed to compile and publish more than a small fraction of these valuable data. Meanwhile, many amateur birders have managed to painstakingly obtain numerous records of Harpy Eagles, many of which are available from online databases. Such databases have become extensive and provide considerable, often underutilized, information. Could a combination of citizen science and published scientific data therefore result in a major advance in an SDM for Harpy Eagles?

Here, we investigate two related topics in Harpy Eagle ecology and conservation: (1) we develop SDMs throughout the species range to identify strongholds and ecologically-suitable areas. We do so by generating and testing the SDMs using environmental variables that are directly linked to Harpy Eagle ecology, which can help produce better conservation policies; and (2) we use these SDM to identify suitable reintroduction sites in the Atlantic Forest. SDM maps can help identify new field sites for future surveys, help create new protected areas specifically designed to conserve Harpy Eagles, and identify marginal or suboptimal habitats as well as potential reintroduction sites. All of these results can help improve conservation policies for the world’s largest eagle.

## Methods

### Data collection

We compiled occurrence records using two main methods: standardized literature searches from Google Scholar and birders’ records at WikiAves (www.wikiaves.com.br). At Google Scholar, we used scientific and vernacular names of the species (in Portuguese, English and Spanish) to look for papers that may contain geographic data. We relied on geographic coordinates provided by authors, but occasionally only maps were available, because some researchers believe that nest sites should remain undisclosed to avoid loss of chicks to wildlife traffickers. When we were unable to contact the authors, we extracted coordinates directly from the maps, but for records that included maps that were not sufficiently precise, we excluded those records. The WikiAves data retrieval was done up to 2016, with records spanning any date. To determine the location of a documentary photo or sound recording, we used municipal county (*município*) information from WikiAves in addition to the location description, consulting the author whenever necessary. Data were double-checked for pseudo-replicates, meaning that we use only one confirmed record for specific nests or individual eagles that had been photographed by multiple birders. We also searched the following georeferenced sound and photo databases: www.birdforum.net, www.xeno-canto.org, and www.macaulaylibrary.org. All records and their geographic coordinates can be found in S1 Table.

### Breeders

Harpy Eagles are selective in their nest tree choice and almost exclusively nest in giant “T-shaped” bifurcations of emergent trees providing a stable platform [47,48]. Those trees used for breeding are of direct interest to the timber industry and are now absent from vast tracts of Amazonian logged forest [49]. We therefore distinguished records of breeding and non-breeding individuals as animals in logged landscapes may not be able to reproduce given the absence of appropriate trees. We concluded that there was evidence of breeding if any of the following conditions were met: (1) eagles with greyish-white plumage, as such eagles are fledglings that are known to be unable to traverse flight distances longer than 2-km from their natal tree [15,16]; (2) adult individuals with brown breast coloration, which can only result from weeks of contact with tannin-rich leaves of the fresh nest material branches during incubation, and then brooding of the young chick [50]; and (3) any individual recorded at a nest. Consequently, we were able to identify locations that were in fact breeding sites for this eagle.

### Databases

For our SDMs, we used remotely sensed large-scale metrics as environmental variables. Specifically, we used data on bioclimatic variables and elevation [51], human population density [52], enhanced vegetation index, which is a measure of the amount of vegetative greenness (and canopy cover [53], and canopy height [54]. All environmental variables had a 1-km^2^ resolution, and analyses were cut to fit our study area, namely the Americas south of 40°N latitude.

### Species distribution modeling

To calculate the species distribution model, we followed three consecutive steps similar to the procedure of “random selection with environment profiling” [55]. First, we performed a rough classification of “suitable” and “unsuitable” habitat areas using an on-class support vector machine. To calculate this area, we set the condition that 90% of the observations must be within the suitable area, a procedure that has been shown to increase overall model discrimination [55]. Pseudo-absences were selected from the “unsuitable area”. However, this sample was not random. We were concerned that detection of Harpy Eagles may be positively correlated with human population density, because detection may be inflated in an area simply due to the presence of more human observers. To minimize the bias on our model estimates, we selected pseudo-absences, giving weights for each cell, with weights proportional to the human population density of a given cell. In this manner, as the bias is present in both presences and pseudo-absences, it would not affect the model outcome [56]. We created as many pseudo-absences as our number of actual observations. Most of the models used here performed best when presented with an equal number of pseudo-absences and presences [57]. A direct test for the presence of bias on our models is in S1 Text.

After pseudo-absences were sampled, we ran multiple environmental models: BIOCLIM, MAXENT, multivariate adaptive regression splines, logistic regression, generalized additive model, random forest, and support vector machine (SVM) networks, a machine learning approach. With this selection, we attempted to select most families of models, namely climatic envelopes, maximum entropy, splines, linear models, classification tools and SVMs. Since some of these models are sensitive to collinearity, we excluded bioclimatic variables that were correlated with one another. To do so, we ran a principal component analysis on the environmental values of our observations and pseudo-absences. We then scanned the variables in descending order of their eigenvalues. If a variable was not correlated by >0.7 with a previously-selected variable, it was retained in the model. With this procedure, we reduced our variable list to: seasonality of temperature (BIO4), annual precipitation (BIO12), precipitation in the coldest quarter of the year (BIO19), precipitation in the warmest quarter (BIO18), precipitation in the driest quarter (BIO17), mean temperature of the wettest quarter (BIO8), mean diurnal temperature range (BIO2), enhanced vegetation index, canopy cover, and canopy height. Using linear models, we added variables only as main-effects model, as GAM models failed to converge if they contained interactions. In all models, we reserved 20% of our observations and pseudo-absences to test the models. We then used the model to predict the quality of each cell in the study area. The third step was to combine all of these prediction maps. We used a weighted average, whereby each map was weighted by its Area Under the Curve (AUC) values. Weighted average was indicated as a robust method for building model consensus [58].

We were concerned that many of these observations did not relate to reproductive individuals, so we added a new step to the analysis. We performed again all three of the previous steps, but this time using only observations of Harpy Eagles that prove breeding is occurring. The results of this new analysis were then combined with all samples (including records of eagles that had been shown to be breeding and those of eagles that showed no sign of reproduction). We considered that for an area to adequately support sustainable Harpy Eagle populations, it must be close enough to an area suitable for its reproduction. To represent that, we drew a circle around each cell that showed suitability for reproduction, and the area of the circle was equivalent to the mean home range size of a typical Harpy Eagle pair. Using the same logic with a continuous metric of habitat quality for reproduction, we used a Gaussian blur on the reproductive predictions with a standard deviation of 25000/1.96 km. This value was chosen considering that a home range area equals 95% of an individual eagle’s total range of movements [59], and that home ranges of Harpy Eagles are approximately 25 km^2^ [60,61]. Merging different distribution models for different activities has been successfully used for California Condors (*Gymnogyps californianus*), showing robust predictive ability [62]. Once the final distribution was set, we used the criteria of equal sensitivity and specificity to categorize habitat quality as either “presence” or “absence” [63].

## Results

**Fig 2:**
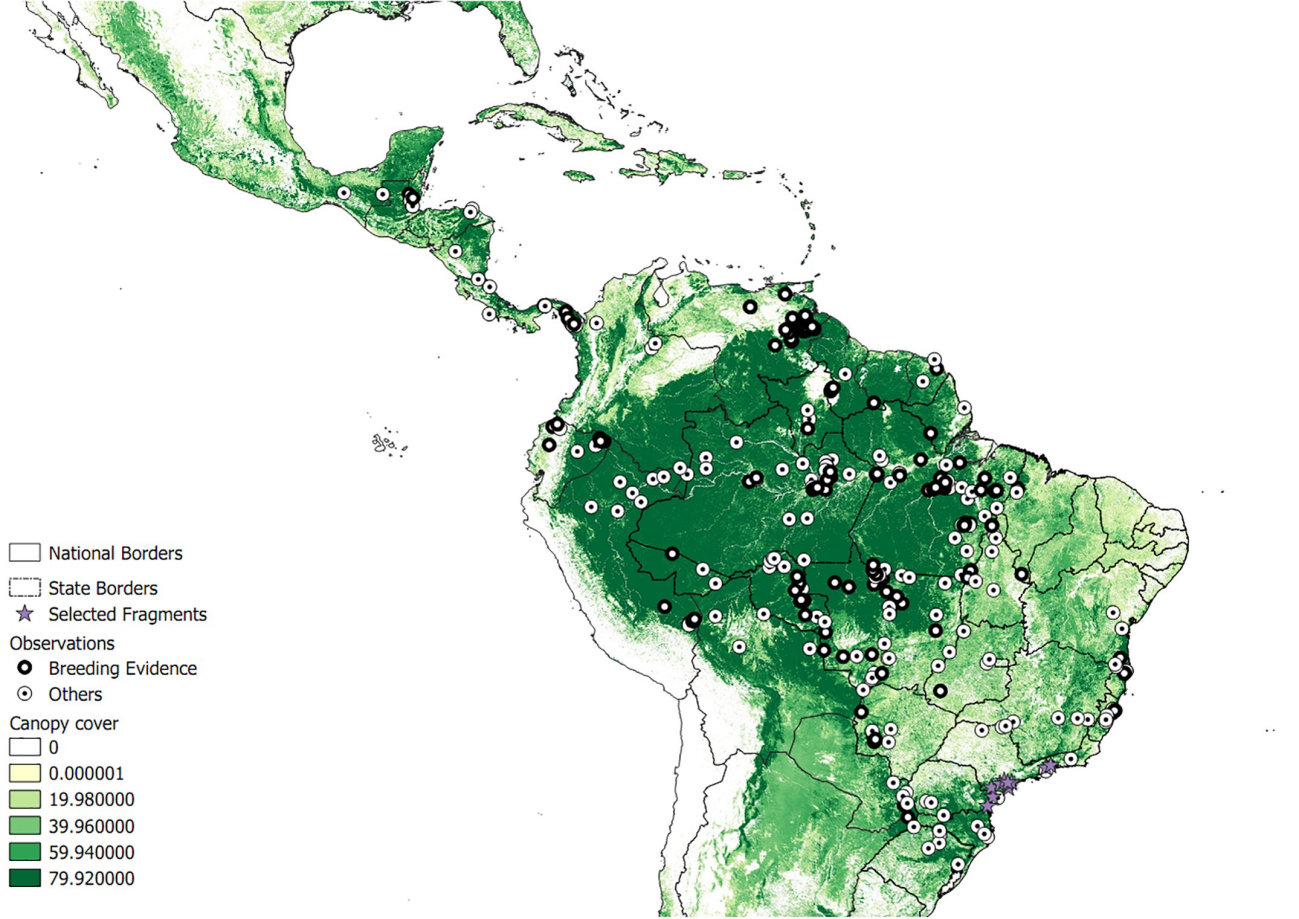
Spatial distribution of the 322 breeding (black circles) and not breeding (white circles) records of Harpy Eagles in Central and South America. Forest cover is shown as a green scale gradient from white (no forest) to dark green (tall canopy forest). Lines represent political country boundaries, and in the case of Brazil, state boundaries. Purple stars are potential reintroduction sites (i.e. predicted suitable habitat areas at present lacking Harpy Eagle populations).

We obtained records of Harpy Eagles with geographic references for all 19 countries that encompass their historical distribution. These include a total of 322 occurrences, 174 records of which consist of individuals that offer no clear evidence of breeding, while 148 records showed evidence of breeding. The largest number of records came from WikiAves (121), followed by scientific articles (118), unpublished theses and dissertations (49), governmental reports (17), birdforum.com (13), and 4 records from miscellaneous sources. According to the AUC values, all models yielded higher predictive power than random models (AUC range for non-reproductive models: 0.7553 (BIOCLIM) to 0.8867 (SVMs); AUC range for reproductive models: 0.7731 (BIOCLIM) to 0.8849 (SVMs, Table 1)). The distribution of those records and the overall potential geographic distribution of Harpy Eagles can be seen in Fig 2.

**Table 1:** Area under the curve of several isolated models, the consensus between them, and the final model, where “All” refers to all records and “Reproductive” refers to records with credible evidence of breeding.

Model	Data	Isolated models	Consensus	Final
BIOCLIM	All	0.7547	0.7788	0.8414
GLM	All	0.8224		
GAM	All	0.8429		
MARS	All	0.8099		
RF	All	0.7698		
MAXENT	All	0.8381		
SVM	All	0.8416		
BIOCLIM	Reproductive	0.7189	0.8026	
GLM	Reproductive	0.8549		
GAM	Reproductive	0.7163		
MARS	Reproductive	0.8846		
RF	Reproductive	0.8657		
MAXENT	Reproductive	0.8491		
SVM	Reproductive	0.9029		

**Fig 3:**
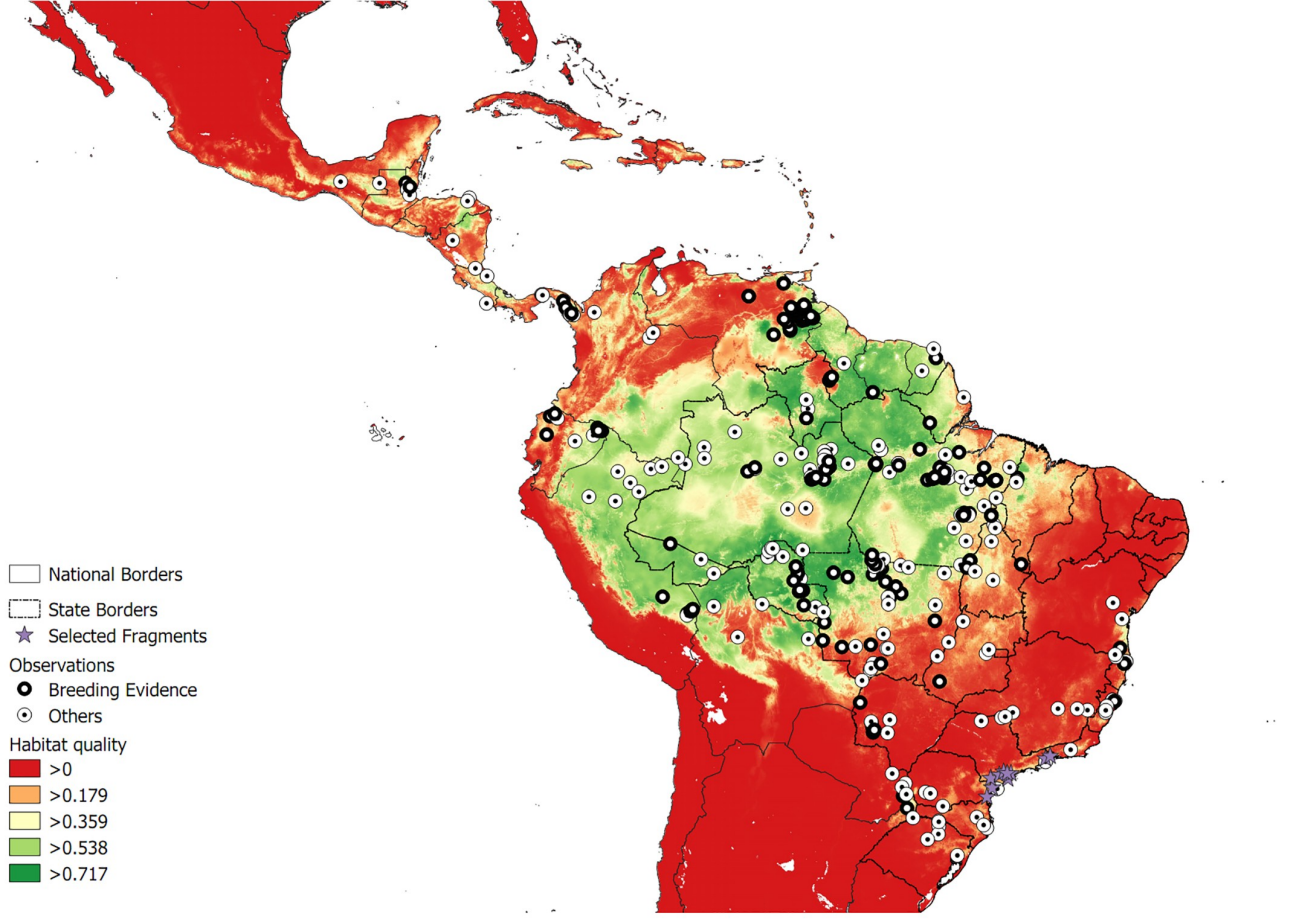
Prediction of the current geographic distribution of conditions fitting the ecological demands of Harpy Eagles in Central and South America under contemporary forest cover. Records of breeding (black circles) and not breeding (white circles) are also shown. The areas considered to be suitable habitat at present are shown in dark green, and uninhabitable areas are shown in red. Purple stars represent suitable reintroduction sites (i.e. predicted suitable habitat areas at present lacking Harpy Eagle populations). Lines represent political country boundaries, and in the case of Brazil, state boundaries.

**Fig 4:**
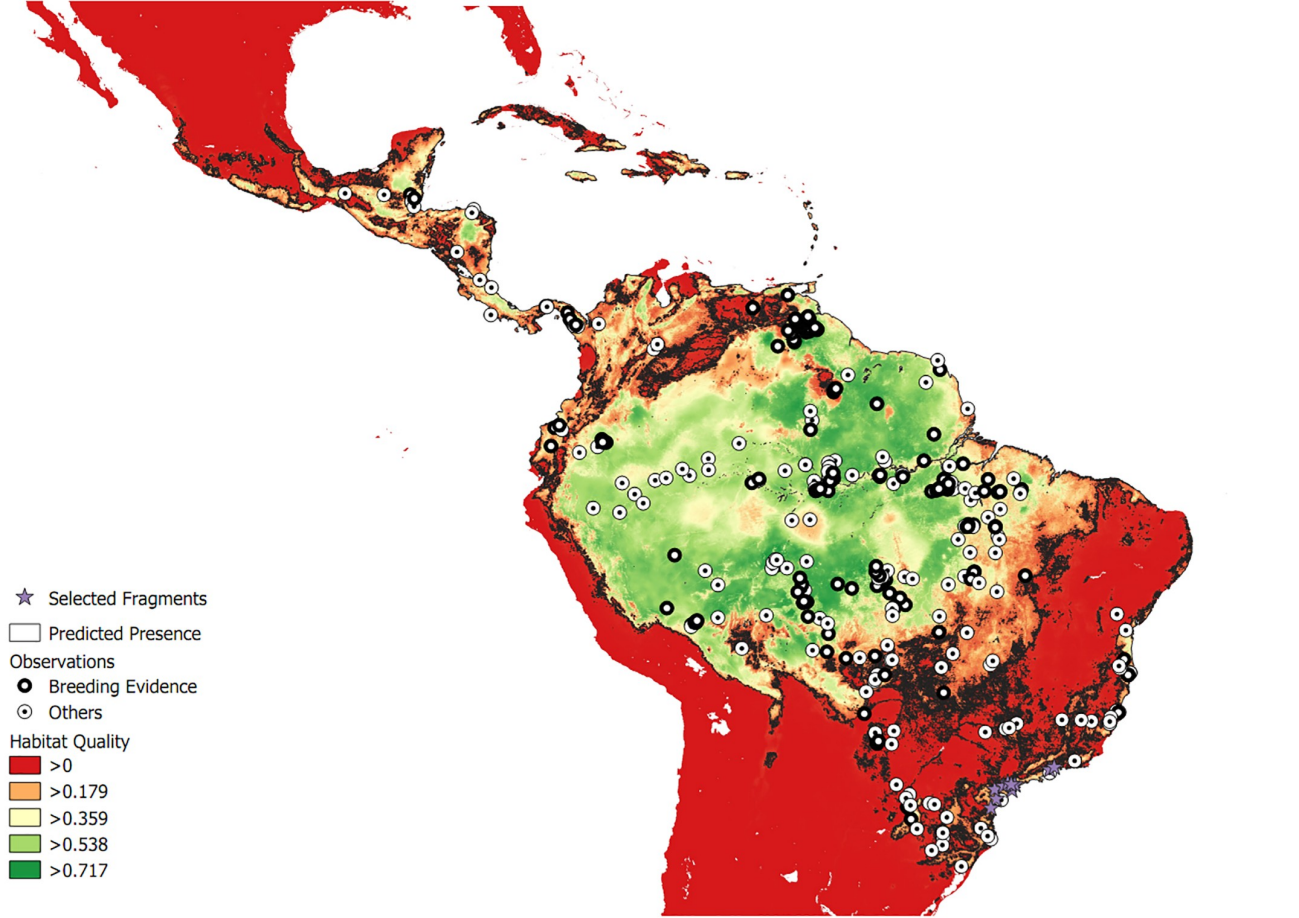
Categorical prediction of the current geographic distribution of conditions fitting the ecological demands of Harpy Eagles in Central and South America under contemporary forest cover (see Methods section for thresholds criteria). Black lines represent the limit between predicted presence and absence. Records of breeding (black circles) and not breeding (white circles) are also shown. The areas considered to be suitable habitat at present are shown in dark green, and uninhabitable areas are shown in red. Purple stars represent suitable reintroduction sites.

The predicted distribution of Harpy Eagles throughout the Neotropics is shown in Fig 3. The model suggests that the Amazon forest is still the largest stronghold for the species, with a continuous area comprising 93% of all currently available habitat (Fig 4). The northern *cerrado* scrubland to wooded savanna macromosaic, mainly located in Brazil’s state of Tocantins, has an extensive patch of intermediate quality Harpy Eagle habitat. Important habitat pockets remain in Mesoamerica, including southeastern Panamá near the Isthmus of Darien, the mosaic of protected areas that straddle Nicaragua and Honduras, and the Selva Maya protected areas that stretch across southern Mexico, Belize and Guatemala.

**Fig 5:**
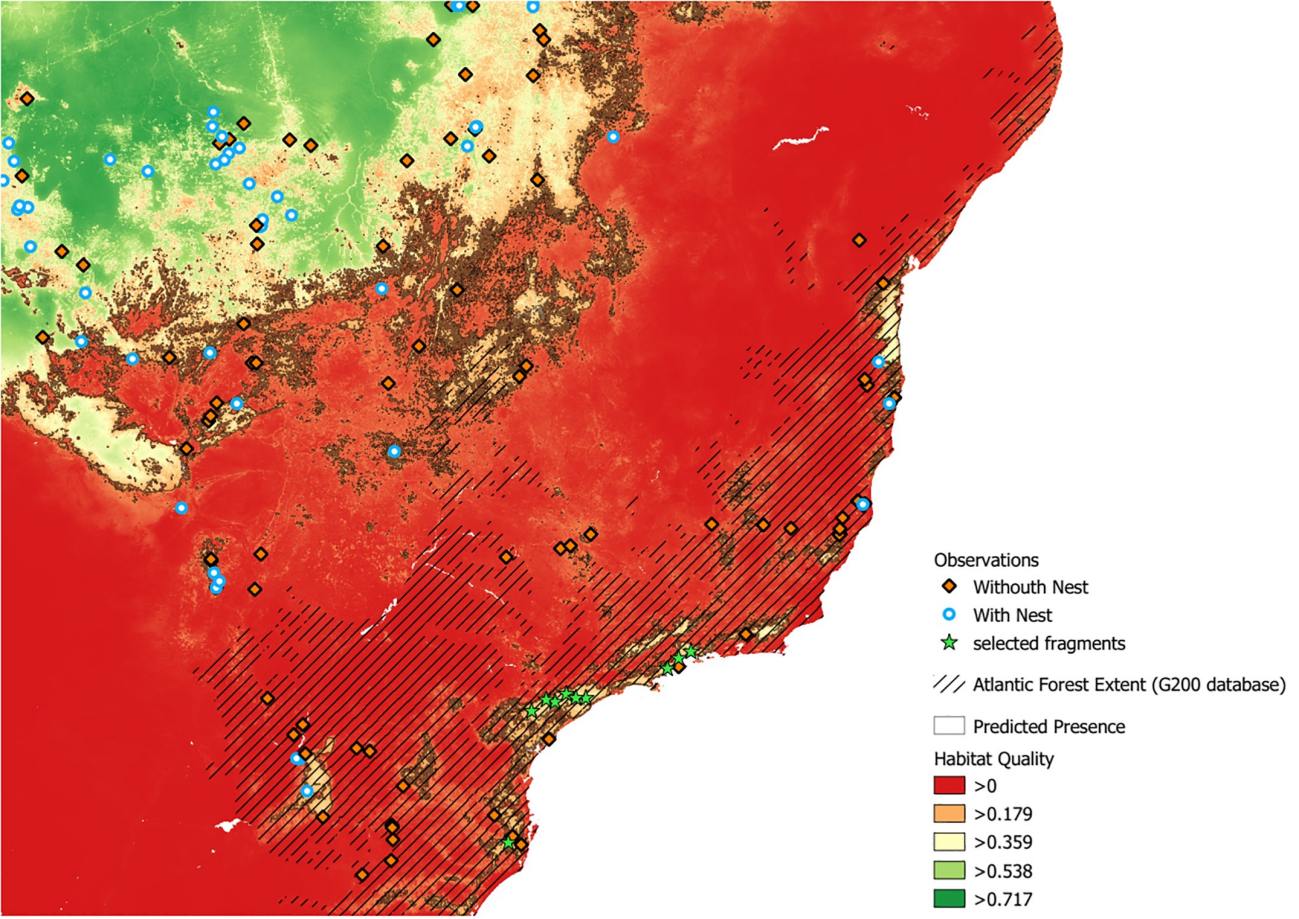
Prediction of the potential Harpy Eagle reintroduction sites in the Atlantic Forest, showing the largest forest fragments that could bear reintroduced populations (i.e. predicted suitable habitat areas at present lacking Harpy Eagle populations), pointed by green stars. They are mainly located along the protected areas of Serra do Mar.

The hyperfragmented landscape of the Atlantic Forest biome retains few available remaining habitat pockets that could currently support viable Harpy Eagle populations. One of them, in the lowland coastal forests of Brazil’s northern Atlantic Forest (in the states of Espírito Santo and southern Bahia) has yielded recent evidence of current populations, including breeding pairs. The other, in northeastern Argentina (Misiones Province), has evidence of breeding in the last decade and recent records of non-breeding individuals. Finally, a ~7,000 km^2^ cluster of forest habitat patches in a large mosaic of coastal protected areas—on the southern section of the Serra do Mar (Fig 5)—potentially shows the best area for future reintroduction attempts across the Brazilian Atlantic Forest. Yet for several decades, this region has yielded no confirmed records of Harpy Eagles.

## Discussion

Careful selection of sites for reintroductions is key to successful species conservation and restoration. Here we delineate for the first time the plausible, global-scale distribution of Earth’s largest extant eagle, a result that is of prime management and conservation interest. Three relatively-small sections of the Atlantic Forest biome demonstrate good habitat suitability for Harpy Eagles, namely: (*i*) the lowlands of the northern Atlantic Forest in Brazil; (*ii*) the Misiones green corridor of Argentina, and (*iii*) the Serra do Mar region of southeastern Brazil. Indeed, the Serra do Mar region has no current records of Harpy Eagles but could host successful reintroduction programs, while other suitable Atlantic Forest sites have recent or current evidence of breeding populations. The Serra do Mar forest corridor could host reintroduced populations that could become viable in the long term, much like the case of the Harpy Eagle reintroduction into Mesoamerica [64]. In contrast, the Amazon Forest currently has extensive tracts of high-suitability forest habitat, mainly concentrated in Brazil, eastern Peru and northern Bolivia. Additional vast tracts of well-suited habitat lie in southeastern Colombia, the Sierra Imataca of eastern Venezuela, and in Guyana, Suriname and French Guyana. Ecuador shows two pockets of suitable habitat, both east and west of the Andes. Mid-elevation tropical Andean forests above 1000 a.s.l. apparently provide suboptimal habitat for Harpy Eagles. Finally, our proposed distribution of over ten million square kilometers (10,401,993 km^2^) represents a reduction of 41% of the neotropical distribution area of 17,600,000 km^2^ that is currently proposed by IUCN [39].

A shortcoming of our methods is that it is not possible to obtain an even distribution of records, leading to some remote regions of the Amazon forest with few records of Harpy Eagles. Those are regions where they certainly occur, whereas our model shows a limited quality habitat section. However, we added two layers of corrections, the random selection with environmental profiling and selecting pseudo-absence using a sampling proportional to the human population. We consequently believe to have constrained this limitation adequately. If going further than that on additional corrections, we could incur in the opposite problem where we give a disproportionally large weight for samples in pristine locations, leading us to falsely conclude the species has stronger conservation requirements than it has indeed. Having recognized and addressed that issue, we will hereafter focus on meaningful elements of the results and in understanding their implications.

### Harpy Eagle range

The Amazon has long been considered the Harpy Eagle’s last stronghold [39,65], and 93% of the current Harpy Eagle range is indeed encompassed by the Pan-Amazonian region. When we attempt to examine the status of Harpy Eagles in what we presume to be its primary Amazonian stronghold, we can simplify the analysis by looking at three broad areas of concern: (A) food, (B) habitat, and (C) mortality.

(A) Regarding the question of an adequate prey base, bushmeat hunting and the resulting competition with humans is a minor issue. Harpy Eagles feed primarily on sloths [66], which in addition to being abundant [67,68], are of minor importance as game species [69]. The effects of secondary forest, hunted or highly degraded forests on the foraging ecology of Harpies remains an open question because to date few has been published on their diet in unhunted, primary forests [70]. Recent observations in Mato Grosso, Brazil, and Sierra Imataca, Venezuela, suggest that this mega-raptor fares well in secondary forest landscapes as long as it is not hunted by local people. Therefore, competition with humans over wild prey is hardly a problem. The ability of these eagles to feed their young with wild prey—chiefly sloths—even in otherwise-hunted landscapes [60,71,72], suggests that Harpies are able to coexist well with humans.
(B) Concerning habitat, the extensive section of degraded forest that we found in much of the southeastern Amazon poses two problems regarding the “last stronghold” assumption: (1) habitat loss by deforestation and (2) habitat degradation by logging and wildfires. The cattle ranching frontier along the Arc of Deforestation continues to advance [73,74], and has already destroyed 23% of all primary terra firme forests of Amazonia. This impact has already led to a reduction in the genetic diversity of Harpy Eagles in this region [41]. Brazil’s recent economic and political crisis and the massive decline in funding directed towards prevention of deforestation, combined with widespread relaxation of environmental laws, has effectively resulted in an unprecedented renewed increase in forest loss [74]. Up to 19,000 km^2^ of primary Amazon forest becomes highly-graded each year by mechanized timber extraction [75], removing low-density giant emergent trees that Harpy Eagles require for nesting. Felling of nest trees by loggers is also a direct source of mortality of eagle chicks [60,76]. The relentless advance of cattle pastures was responsible for another 7,900 km^2^ of forest loss in 2018 alone, which is increasing since 2012 [77]. Population densities of Harpy Eagles have been estimated at only 3–6 nests per 100 km^2^ [78], thereby reiterating the crucial need for megareserves in Amazonia [79].
(C) Eagle killings by humans is another serious issue in the Amazon [20]. Amerindian reserves cover approximately 27% of the Brazilian Amazon [80]. In these Amerindian reserves, Harpy Eagles are universally considered to be prized birds for headdresses and arrow fletching [13,22]. Whereas indigenous societies may have gradually acquired a dynamic equilibrium with the wildlife that remained following the Pleistocene extinctions [81], the acquisition of firearms by Amerindians places much greater powers of destruction in the hands of indigenous people throughout the Amazon [82]. Native Amazonians wielding firearms, combined with the high prices commanded by indigenous feather headdresses when sold illegally as handicrafts, has greatly increased the pressure on Harpy Eagle populations inside Indigenous Lands. Although we are clearly in favor of indigenous land rights, sustainable use of wildlife often fails within indigenous territories and extractive reserves [83–85]. The discussion about hunting of threatened species cannot be trivialized or swept under the rug using the clichéd term “traditional practice”. Rather, sensible rules and bag-limits, if any offtake can be defined as sustainable, as well as effective law enforcement are required to prevent endangered wildlife from melting away through careless use by communities who are directly connected to outside markets. Furthermore, when government land reform agencies settle millions of poor socio-economic migrants in primary Amazonian forest [86], the settlers tend to shoot in rapid succession every Harpy Eagle as well as other large, diurnal raptors [17,18,87].

The Harpy Eagle’s “last stronghold” is therefore far from an adequate safety net, as they are caught in the crossfire generated by market-integrated indigenous groups, high-grading loggers, land reform settlers, and cattle ranchers. These threats should therefore convince IUCN board-members to reassess the conservation status of the Harpy Eagle.

The occasional occurrence of Harpy Eagles in some marginal habitats has been the subject of some discussion [44,45,88]. While early naturalists recorded this species in the Cerrado of central Brazil [65], Harpies were apparently never abundant in this ecosystem. The eagle’s strong preference for giant, T-shaped emergent trees for nesting [47,48], and their specialized feeding habits concentrated on sloths (which are absent outside tropical forests) should render the Cerrado a marginal habitat for this species. Perhaps because of this, many maps show an erroneously disjunct distribution for the species with two separated pockets in South America, excluding the savanna regions between them. This is the case of IUCN map, which makes our proposed 41% reduction in range size even more shocking. Our results suggest that a pocket of acceptable Harpy Eagle habitat exists in the northern Cerrado and in much of the transition zone between the Cerrado and the Amazon, which could explain occasional reports of individuals shot and nests found in such areas. In the Pantanal wetlands, our SDMs suggests that this species is expected to occur only in its northern parts (with very limited habitat quality and range), where the few direct records have been documented for the species [44]. An extensive search of the entire Pantanal for the similarly-huge nests of Jabiru storks (*Jabiru mycteria*) found no Harpy nests whatsoever, suggesting absence [89]. A couple of Harpy Eagles have been recently documented at the Calileuga National Park in the Yungas of northwestern Argentina, which contains a small habitat patch that our SDM shows to be of low quality. Another peripheral habitat area that shows several pockets of good suitability are the Caribbean Antilles. It is interesting to note that none of the bird-rich fossil records of Antillean islands have uncovered any remains of Harpy Eagles. Several species of giant raptors that humans drove to global extinction are known from this archipelago [90,91]. These extinct predators include a giant flightless owl (*Ornimegalonyx oteroi*), a giant flying owl (*Tyto pollens*) and a giant, buteo-type hawk (*Amplibuteo woodwardi*). It would be interesting to investigate if those extinct Antillean raptors performed a similar predation role on both terrestrial and arboreal sloths of the Antilles as Harpy Eagles exert on arboreal sloths in continental forest ecosystems. These musings open many interesting lines of inquiry regarding convergent predator-prey relationships in Caribbean islands and continental Neotropical forests.

### Atlantic Forest reintroduction

Range models can be interpreted as related to environmental suitability for the target species, where higher index values suggest better habitat conditions [92][93]. The Harpy Eagle’s best sections of remaining habitat in the Atlantic Forest biome primarily consist of high-stature, lowland forest. One of these sections is the region that harbors some of the last breeding pairs of the species in the Atlantic Forest, specifically in the forest reserves of Sooretama, Linhares, Serra Bonita, Descobrimento, and Pau Brasil [28,29,94]. Over the last five centuries, Atlantic Forest landscapes have become highly degraded by conversion into sugarcane, coffee, and cacao plantations, slash-and-burn agriculture, and timber extraction [95], followed by extensive cattle ranching and eucalyptus monocultures, the latter two of which tolerate the resulting nutrient-poor soils. Thus, the Atlantic Forest has been an epicenter of forest loss in South America, beginning several centuries prior to the consolidation of the “Arc of Deforestation” in the southern, eastern and southeastern Amazon [96]. After centuries of various direct sources of forest depletion, the Atlantic Forest currently presents small—but still worthwhile—hotspots for Harpy Eagle conservation. A highly-biodiverse, shade-grown-cacao-based economy [97] can still host successful conservation programs in the northern Atlantic Forest [98], and that includes Harpy Eagles. At the other extreme of the land use spectrum, the strictly-protected reserve network in the Serra do Mar forest corridor could provide promising habitat for a “rewilding” reintroduction project that would rebuild long disrupted forest trophic cascades in the southern Atlantic Forest. We recommend that conservationists planning significant reintroduction efforts for Harpy Eagles and other apex predators consider the findings from our models. We also emphasize that the parks within the Serra do Mar Atlantic Forest region should be given highest priority for release sites if any rehabilitated individuals become available near or in the Atlantic Forest.

A key factor regarding site selection in the Serra do Mar Atlantic Forest, where we recommend reintroductions, is that a sizable portion of this region falls outside the distribution of sloths in the Atlantic Forest [99]. In the absence of sloths, Harpy Eagles may take a disproportionately high toll on other arboreal mammal prey species, such as capuchin monkeys. In the Serra do Mar, capuchins have densities as high as 32 individuals per km^2^ [100], and these monkeys are known to seasonally decimate threatened arborescent palms [101]. Problems related to capuchins crop-raiding forest plantations have also been reported elsewhere in the southern Atlantic Forest [102], where reintroduced Harpy Eagles could regulate monkey populations [32,33]. In the southern Atlantic Forest, remarkable work to connect fragmented landscapes is being carried out for Jaguars [103], and this model could be replicated for Harpy Eagles. Cross-fertilization between research programs for both of these top predator species could provide highly positive synergistic outcomes. Reintroductions have become a central focus of attention in the Atlantic Forest conservation agenda [104,105]. Reintroductions of top predators must, however, take into consideration issues related to a number of threatened arboreal mammals of the Atlantic Forest. Blonde Capuchins (*Sapajus flavius*; [106]), Maned Sloths (*Bradypus torquatus*; [107]) and Bristle Porcupines (*Chaetomys subspinosus*; [108]) are just a few examples of endangered species that may be further imperilled by reintroduced predators, as has been shown elsewhere [109]. Fortunately, none of those prey species are in the set of regional sites where we propose reintroducing Harpy Eagles.

In Brazil, at least several conservation breeders have successfully reproduced Harpy Eagles (e.g. Bela Vista Biological Refuge, Roberto Ribas Lange Zoo and CRAX). Each of these breeders hold over several adult individuals, and other private breeders have a smaller number of adults and young animals totaling several dozens in Brazil [110]. Meanwhile, The Peregrine Fund has developed a huge amount of know-how on Harpy Eagle reintroductions during a directed restoration effort for the species in Mesoamerica [64,111–113]. In addition, the Brazilian Harpy Eagle Conservation Program successfully released several rehabilitated individuals [61]. Eliminating the causes of extirpation must be addressed before embarking on any reintroduction effort in the Atlantic Forest. Given that large remaining portions of the historically-overexploited Atlantic Forest are no longer losing additional forest, the main threat to reintroduced Harpies would be reprisal or prophylactic killings by local residents [20,64]. Harpy Eagles also present a unique opportunity for ecotourism development that has shown positive results for both predators and local economies when implemented in a controlled, responsible manner [114,115]. Therefore, given the current amount and high quality of expertise, we believe that if appropriate funding can be raised, a successful reintroduction effort can become feasible.

In conclusion, we show that in the Amazon forest—the Harpy Eagle’s last stronghold—much of the forest that could be considered prime habitat for the species may in fact already be badly degraded by the rapidly-expanding Arc of Deforestation and associated logging frontiers. Regarding reintroductions at the Atlantic Forest, the most suitable sites for Harpy Eagle are located in the Serra do Mar forest corridor. In the currently hyperfragmented landscapes of the Atlantic Forest, this habitat corridor represents the largest tropical forest continuum available that could host a healthy population of Harpy Eagles. Much of this forest corridor lies within protected areas that could support a reintroduction project for Harpy Eagles, so environmental authorities should prioritize this corridor as a release site for Harpies. Here we sound the alarm that the supposedly uniformly high-quality of Amazonian forests as a long-term refuge for Harpy Eagles is far from ideal. Rather, a perverse mix of anthropogenic threats has been driving Harpy Eagles to local extinction long before the forest cover is completely removed. We therefore suggest that in light of these findings, the IUCN status of this keystone predator should be reassessed.

## Supporting information

S1 Table Harpy Eagle occurrences. Contains all records and their geographic coordinates that were used to build the paper.
(XLSX)

S1 Text Testing for bias in range modelling. Contains a detailed description of a direct test for the presence of bias on our models.
(PDF)
