# Supplementary material for: Species distribution modeling reveals strongholds and potential reintroduction areas for the world’s largest eagle
Source: PLoS One. 2019 May 13;14(5):e0216323. doi: 10.1371/journal.pone.0216323 (PMC6513255; doi:10.1371/journal.pone.0216323)
Supplement: S1 Text — Contains a detailed description of a direct test for the presence of bias on our models. (PDF) [file pone.0216323.s002.pdf]

Testing for bias in the environmental niche modelling of Harpia harpyja

Supplementary material of: *Ecological niche models reveal potential areas for Harpy Eagle reintroduction in Atlantic Forest*

This Supplementary material aims to test whether there is a bias in the samples for population occurance. More specifically, if areas with higher human population density have more samples than areas with lower population samples. We could expect such bias because areas with little human presence are unlikely to have documented evidence of harpies, since there is less people to spot and report our study animal. In the interest of making this proof as reproducible as possible, we present this analysis results and code in a R markdown format.

We begin by loading data. For this analysis we will use the quality map calculated by the ENM (i.e. the probability of occurance), the population dataset from the gridded population of the world (GPWv4). Population is measured as number of people per km^2. In addition, we also use the train and test dataset. This dataset is presented as a spreadsheet (a data.frame in R terminology) with each row representing a absence or presence. Columns represent environmental variables and a binary representation of presence-absence (i.e. 1 equals to presence, 0 equals to absence). We call the latter quality.

```
library(knitr)
opts_chunk$set(tidy.opts=list(width.cutoff=20),tidy=TRUE)
setwd("D:\\data jorge computer lab\\ENM Harpies - Jorge\\ENM Harpies - Jorge Menezes")
library(raster)
library(mgcv)
quality = raster("./ENM output/misto.tif")
pop     = stack("./Processed environmental/envir_stack.grd")[[23]]
dataset = read.csv(".\\ENM submodels\\data all.csv")
load("./ENM output/model objects.RData")
```

Once loaded, we first question whether there is a significant difference in the population size between where our pseudo-absences and presences were taken. We also ask if there is significant difference in from a random sample. The latter would indicate complete absence of bias related to the population size. However, an acceptable compromise is to that pseudo-absence and presences would have the same bias. If they do, this bias should not influence the calculation of presence or absence since the same bias is present in absence and presence data. We conduct this test below:

```
dist = sampleRandom(pop,
    1000)  # Population distribution on a random sample of distribution
distpa = dataset$n_humans[dataset$quali ==
    0]  # Population distribution on the pseudo-absence points
distpres = dataset$n_humans[dataset$quali ==
    1]  # Population distribution on the presence points
means = c(random.samples = mean(dist,
    na.rm = T), pseudo.absences = mean(distpa,
    na.rm = T), presences = mean(distpres,
    na.rm = T))
print(means)
```

```
##  random.samples pseudo.absences       presences
##        34.12499       100.87697        54.14693
```

```
t1 = t.test(dist, distpa)
t2 = t.test(dist, distpres)
```

```
t3 = t.test(distpa, distpres)
result = data.frame(t = c(t1$stat,
    t2$stat, t3$stat),
    df = c(t1$param,
        t2$param, t3$param),
    p = c(t1$p.val, t2$p.val,
        t3$p.val))
print(result)
```

```
##             t       df          p
## 1 -2.9458857 403.6821 0.00340685
## 2 -0.6973029 362.6298 0.48606005
## 3  1.3437695 580.6272 0.17954782
```

The means of population density are indeed different between each type of data. The difference between random samples and presence samples is significant, but the other two tests are not. This indicates that presence has the same population density than pseudo-absence and the latter cannot be separated from a random sample. Hence we achieve the compromise criterion,and support indirectly the more rigorous criterion (presences are similar to absences which are similar to random). Below a visualization of each distribution with means as vertical lines. Green represents of presence data, red represents of pseudo-absences and black represents of the random distribution.

```
plot(density(distpres,
    n = 1024, na.rm = T,
    from = 0), col = "green",
    lwd = 2, xlim = c(0,
        200), main = "",
    xlab = "Population density",
    ylab = "Density of Studies")
lines(density(distpa,
    n = 1024, na.rm = T,
    from = 0), col = "red",
    lwd = 2)
lines(density(dist, n = 1024,
    na.rm = T, from = 0),
    col = "black", lwd = 2)

abline(v = means[1])
abline(v = means[2],
    col = "red")
abline(v = means[3],
    col = "green")
```

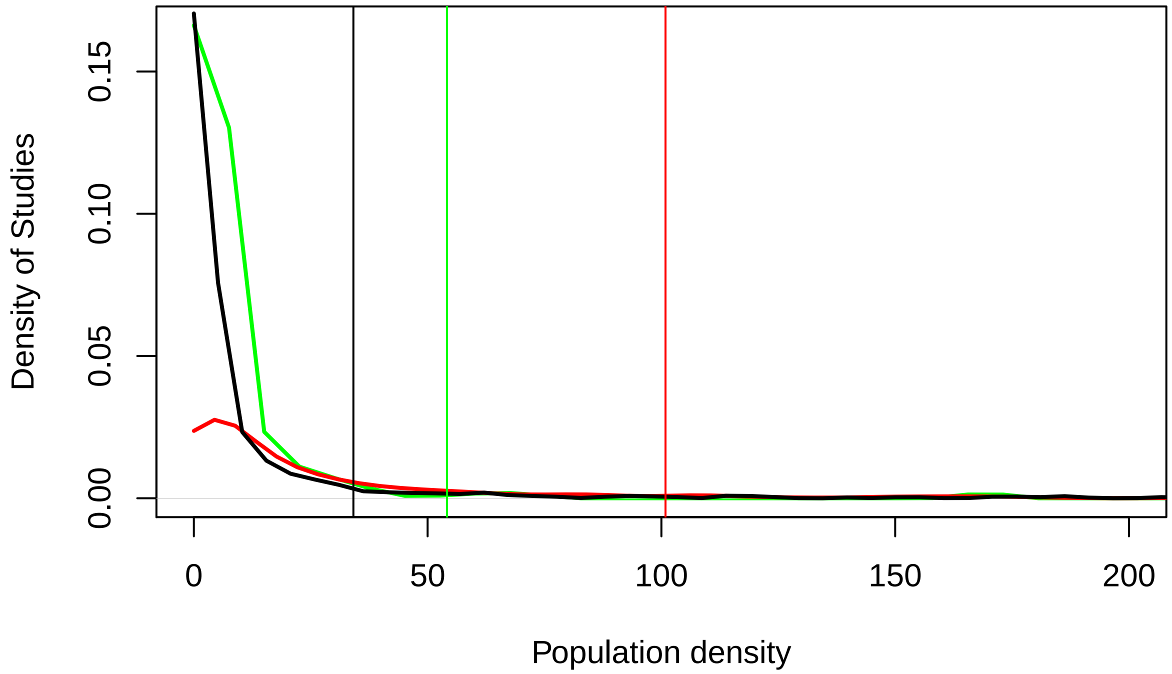

Overall our results indicate no presence of bias. Nevertheless, we also checked in another manner. Our main concern with population bias is that it may lead the model to believe deforestated areas are better than non-deforestated areas, simply because those areas have enough humans to detect and sample harpies. If that was the case, we would see a negative correlation between habitat quality for harpies (probability of occurance according to the model) and canopy cover. To verify that we look in the effect of canopy cover on the probability of occurance calculated by the GLM and GAM models. Both models give estimates that are easily represented, in constrast with models such as maxent and random forest where the effect of one variable is difficult to extract.

```r
summary(mglm)
```

```
##
## Call:
## glm(formula = quali ~ (.), family = binomial, data = train.all)
##
## Deviance Residuals:
##      Min        1Q    Median        3Q       Max
## -2.32014  -0.64113  -0.04476   0.69025   2.06079
##
## Coefficients:
##                 Estimate Std. Error z value Pr(>|z|)
## (Intercept)   -2.078e+00  5.570e-01  -3.731 0.000191 ***
## bio12         -4.925e-05  3.697e-04  -0.133 0.894018
## bio4          -7.262e-04  1.444e-04  -5.028 4.96e-07 ***
## bio19          1.780e-04  6.377e-04   0.279 0.780086
## ndvi           4.295e+00  1.082e+00   3.969 7.23e-05 ***
## bio18         -5.987e-04  8.919e-04  -0.671 0.502110
```

```
## bio17         -1.344e-03  1.291e-03  -1.041 0.297836
## canopy_cover   2.869e-02  5.339e-03   5.373 7.75e-08 ***
## canopy_height  1.970e-02  1.325e-02   1.486 0.137220
## ---
## Signif. codes:  0 '***' 0.001 '**' 0.01 '*' 0.05 '.' 0.1 ' ' 1
##
## (Dispersion parameter for binomial family taken to be 1)
##
##     Null deviance: 692.64  on 499  degrees of freedom
## Residual deviance: 428.58  on 491  degrees of freedom
## AIC: 446.58
##
## Number of Fisher Scoring iterations: 6
```

```r
summary(mgam)
```

```
##
## Family: binomial
## Link function: logit
##
## Formula:
## quali ~ s(bio12) + s(bio4) + s(bio19) + s(ndvi) + s(bio18) +
##     s(bio17) + s(canopy_cover) + s(canopy_height)
##
## Parametric coefficients:
##             Estimate Std. Error z value Pr(>|z|)
## (Intercept)  -1.1179     0.3252  -3.438 0.000587 ***
## ---
## Signif. codes:  0 '***' 0.001 '**' 0.01 '*' 0.05 '.' 0.1 ' ' 1
##
## Approximate significance of smooth terms:
##                    edf Ref.df Chi.sq  p-value
## s(bio12)         4.083  5.000 14.034  0.01535 *
## s(bio4)          1.000  1.000 18.158 2.03e-05 ***
## s(bio19)         7.896  8.639 17.826  0.02445 *
## s(ndvi)          1.000  1.000  5.520  0.01880 *
## s(bio18)         7.711  8.514 13.198  0.17316
## s(bio17)         4.888  5.963  5.770  0.44490
## s(canopy_cover)  2.137  2.670 11.163  0.00825 **
## s(canopy_height) 2.546  3.110  6.357  0.09689 .
## ---
## Signif. codes:  0 '***' 0.001 '**' 0.01 '*' 0.05 '.' 0.1 ' ' 1
##
## R-sq.(adj) =  0.559   Deviance explained = 52.9%
## UBRE = -0.21794  Scale est. = 1         n = 500
```

```r
plot(mgam, select = 7,
    xlab = "Canopy cover",
    ylab = "Partial response")
```

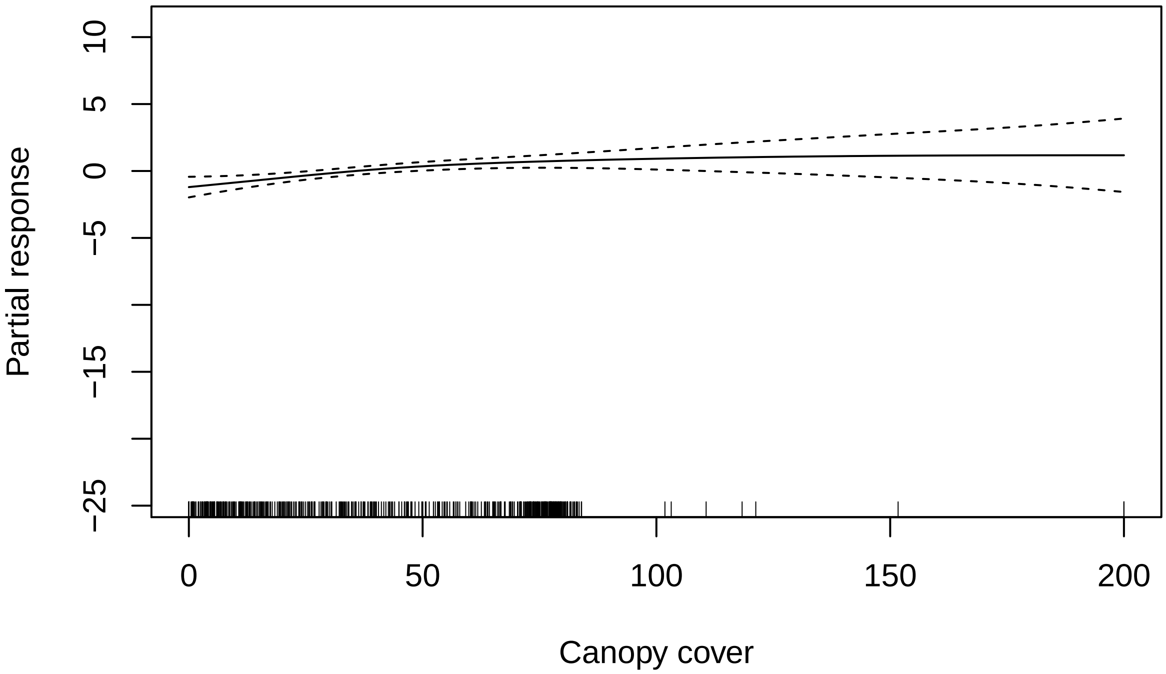

Both GLM and GAM show the effect of canopy cover is small, marginally significant (in other words, we cannot affirm it is different from 0). The visualization of GAM also corroborate this evidence with a slight positive effect. Hence have no reason to believe our data is artificially downsampling regions with high canopy cover.

For a final examination, we also tested if there is any correlation between the quality estimated by the non-reproductive consensus model and population density. We found a negative correlation of -0.0508732 which indicates that if there is such an effect, it is small and in the opposite direction of what would be expect if lack of observation would explaning our data. This is demonstrated below:

```
pop.comp = projectRaster(pop,
    quality)
stack.cor = stack(quality,
    pop.comp)
cor.value = layerStats(stack.cor,
    "pearson", na.rm = T)
```

Considering all our three analysis indicate a non-significant and/or small bias. We conclude that the obsever bias should not explain our current results.
